# Complete Genome Sequence of *Microbacterium* sp. Strain Nx66, Isolated from Waters Contaminated with Petrochemicals in El Saf-Saf Valley, Algeria

**DOI:** 10.1128/MRA.01130-20

**Published:** 2020-11-19

**Authors:** Nardjess Chéraiti, Frédéric Plewniak, Salima Tighidet, Amalia Sayeh, Lisa Gil, Ludivine Malherbe, Yosr Memmi, Laurence Zilliox, Céline Vandecasteele, Pierre Boyer, Céline Lopez-Roques, Benoît Jaulhac, Mourad Bensouilah, Philippe N. Bertin

**Affiliations:** a Laboratoire d’Ecobiologie des Milieux Marins et Littoraux, Université Badji Mokhtar, Annaba, Algérie; b Génétique Moléculaire, Génomique et Microbiologie, Université de Strasbourg, Strasbourg, France; c Laboratoire de Microbiologie Appliquée, Université de Bejaia, Bejaia, Algérie; d Université de Bouira, Bouira, Algérie; e INRA, US 1426, GeT-PlaGe, Genotoul, Castanet-Tolosan, France; f Institut National des Sciences Appliquées et de Technologies, Tunis, Tunisie; g Centre National de Référence Borréliose, Laboratoire de Bactériologie, CHU de Strasbourg-Hôpital Civil, Strasbourg, France; University of Delaware

## Abstract

*Microbacterium* sp. strain Nx66 was isolated from waters contaminated by petrochemical effluents collected in Algeria. Its genome was sequenced using Illumina MiSeq (2 × 150-bp read pairs) and Oxford Nanopore (long reads) technologies and was assembled using Unicycler. It is composed of one chromosome of 3.42 Mb and one plasmid of 34.22 kb.

## ANNOUNCEMENT

Actinobacteria are Gram-positive aerobic bacteria widely distributed in terrestrial and aquatic ecosystems and are known to produce a great variety of bioactive compounds (1, 2). They are mainly free living, commensals, or symbiotic, but some of them may cause infections in humans (3–8).

A total of 28 strains were isolated from a water sample collected in Skikda’s El Saf-Saf Valley, Algeria (36.87981N, 6.93111E), receiving industrial releases from a petrochemical refinery (9). Aliquots of 100 μl up to 10^−3^ dilutions were inoculated onto Reasoner's 2A agar (R2A) agar plates incubated at 30°C for 24 h to 1 week, and bacterial colonies were purified by streaking three times onto fresh medium agar plates. Protein samples were prepared from colonies using a mix (50/50) of 70% (vol/vol) formic acid (Sigma, Lyon, France) and 50% (vol/vol) acetonitrile (Fluka, Buchs, Switzerland) and were analyzed by mass spectrometry as previously described (10). The Nx66 isolate was identified with low confidence as a *Microbacterium* strain.

Nx66 cells were grown for 72 h in R2A liquid medium at 30°C, and DNA was extracted using the MasterPure complete DNA and RNA purification kit (Epicentre). An Oxford Nanopore Technologies (ONT) library was prepared according to the manufacturer’s instructions for 1D native barcoding genomic DNA (kits EXP-NBD103 and SQK-LSK109). DNA was quantified using the Qubit double-stranded DNA (dsDNA) high-sensitivity (HS) assay kit (Life Technologies), and purity was determined using a Nanodrop instrument (ThermoFisher). Size distribution and degradation were assessed using the fragment analyzer (AATI) high-sensitivity DNA fragment analysis kit. DNA was purified using AMPure XP beads (Beckman Coulter) and sheared at 20 kb using the Megaruptor system (Diagenode). One DNA damage repair, end repair, and dA tail step was performed before sample-specific index ligation. The library was loaded on an R9.4.1 revD flowcell and sequenced on a GridION instrument at 0.03 pmol within 48 h using MinKNOW v2.0.10-1 and Guppy v1.8.5-1 for base calling. Illumina 2 × 150-bp paired-end libraries were prepared according to Illumina’s protocols using the TruSeq Nano DNA high-throughput (HT) library prep kit. DNA was fragmented by sonication. Size selection was performed using sample purification beads (SPBs). Library quality was assessed using an Advanced Analytical fragment analyzer. Libraries were quantified by quantitative PCR (qPCR) using the Kapa library quantification kit. Sequencing was performed on an Illumina MiSeq instrument with V2 reagent kits.

Adaptors and low-quality extremities (Q, <20) were trimmed off short reads with BBDuk (https://jgi.doe.gov/data-and-tools/bbtools/bb-tools-user-guide). Read pairs with a Q value of <30 were discarded. Adaptors were trimmed off long reads using the Oxford Nanopore Technologies qcat program (https://github.com/nanoporetech/qcat). Long reads with a Q value of <9 were discarded using Nanofilt v2.5.0 (11). Assembly was performed with Unicycler v0.4.7 (12) using default parameters, yielding two circular replicons of 3,422,870 bp and 34,223 bp with GC contents of 70.17% and 66.07%, respectively. QUAST v5.0.2 (13) rated the assembly as good (mapping reads, 99.62%; coverage, 79× for Illumina and 117× for Nanopore). Analysis with CheckM v1.0.11 (14) returned 99.49% completeness, insignificant contamination (0.51%), and no strain heterogeneity.

**FIG 1:**
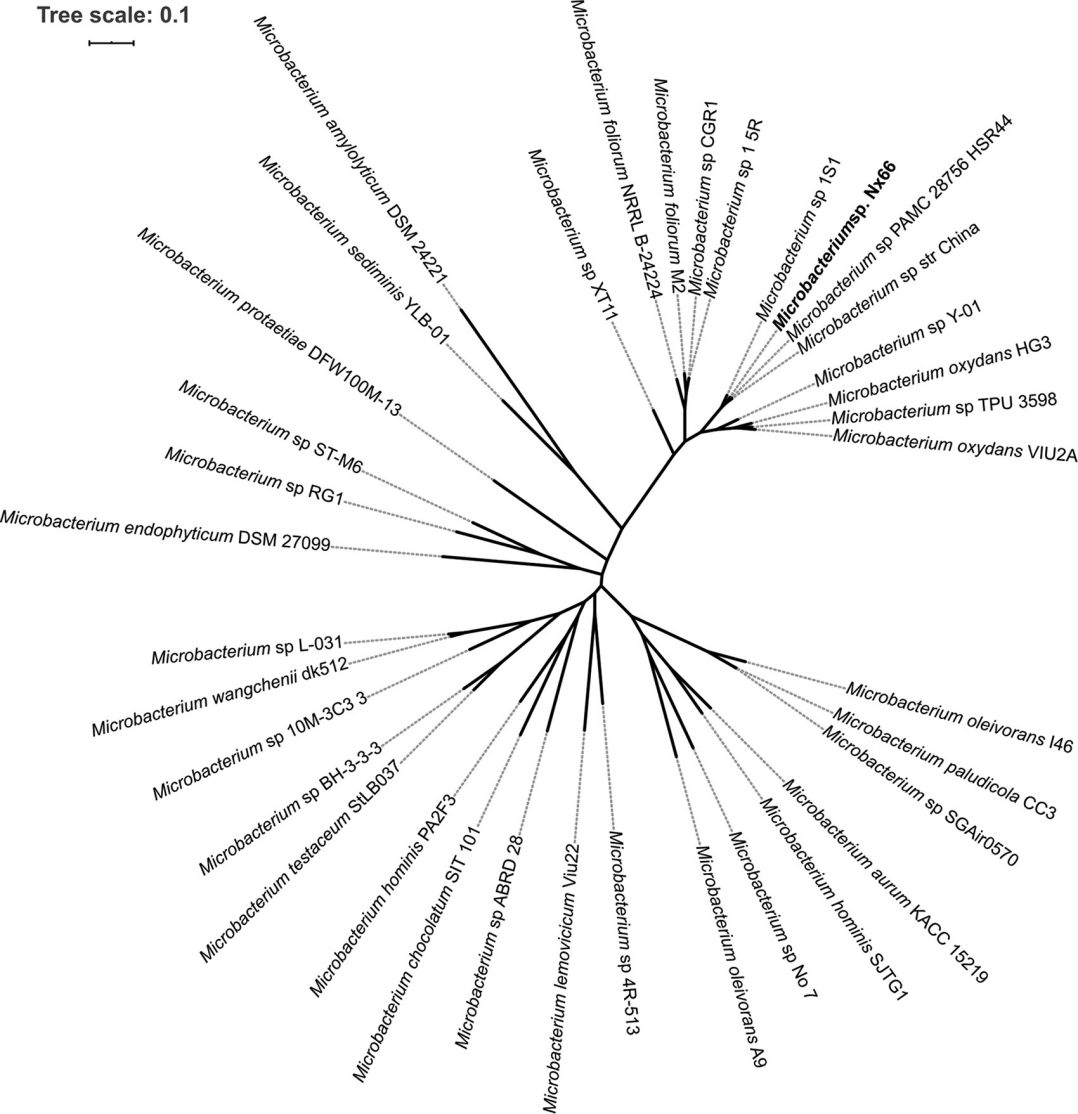
Unrooted maximum likelihood phylogenetic tree of Nx66 and the 35 publicly available *Microbacterium* complete genome sequences. The tree was obtained with GToTree v1.4.16 with HMM source Actinobacteria.hmm (138 targets) and default parameters (JTT+CAT model) (20). The *Microbacterium* sp. Nx66 closest relatives were *Microbacterium* sp. strain China and *Microbacterium* sp. PAMC 28756 HSR44. The 35 complete genomes and their RefSeq accession numbers are Microbacterium amylolyticum (GCF_011046975.1), Microbacterium aurum (GCF_001974985.1), Microbacterium chocolatum (GCF_001652465.1), Microbacterium endophyticum (GCF_011047135.1), Microbacterium foliorum (GCF_003367705.1), Microbacterium foliorum (GCF_006385575.1), Microbacterium hominis (GCF_002843965.1), Microbacterium hominis (GCF_013282805.1), Microbacterium lemovicicum (GCF_003991875.1), Microbacterium oleivorans (GCF_001975955.2), Microbacterium oleivorans (GCF_013389665.1), Microbacterium oxydans (GCF_003991855.1), Microbacterium oxydans (GCF_004000565.1), Microbacterium paludicola (GCF_001887285.1), Microbacterium protaetiae (GCF_004135285.1), Microbacterium sediminis (GCF_004564075.1), *Microbacterium* sp. 1.5R (GCF_001889265.1), *Microbacterium* sp. 10M-3C3 (GCF_003931875.1), *Microbacterium* sp. 1S1 (GCF_008271365.1), *Microbacterium* sp. 4R-513 (GCF_011046485.1), *Microbacterium* sp. ABRD_28 (GCF_003850245.1), *Microbacterium* sp. BH-3-3-3 (GCF_001792815.1), *Microbacterium* sp. CGR1 (GCF_001266755.1), *Microbacterium* sp. L-031 (GCF_008727775.1), *Microbacterium* sp. No. 7 (GCF_001314225.1), *Microbacterium* sp. PAMC 28756 (GCF_001558975.1), *Microbacterium* sp. RG1 (GCF_005347485.1), *Microbacterium* sp. SGAir0570 (GCF_005491085.2), *Microbacterium* sp. ST-M6 (GCF_008727755.1), *Microbacterium* sp. strain China (GCF_002993305.1), *Microbacterium* sp. TPU 3598 (GCF_002356155.1), *Microbacterium* sp. XT11 (GCF_001513675.1), *Microbacterium* sp. Y-01 (GCF_003856715.1), Microbacterium testaceum (GCF_000202635.1), and Microbacterium wangchenii (GCF_004564355.1).

*Microbacterium* genus assignment was confirmed by the RDP classifier (15) using 16S rRNA genes predicted with barrnap (https://github.com/tseemann/barrnap). The best average nucleotide identity computed with FastANI v1.2 (16) against the 35 complete public *Microbacterium* genomes was obtained with *Microbacterium* sp. strain China (93.45%, 1,062/1,151 fragments). This value, lower than observed intraspecies values (17, 18), suggests that Nx66 is close but not identical to strain China (Fig. 1).

Annotation with the MicroScope platform (19) predicted 3,506 genes in the chromosome (3,441 coding sequences [CDS], 47 tRNA genes, and 6 rRNA genes) and 41 CDS in the plasmid. The chromosome annotation showed the presence of a significant number of genes involved in resistance to toxic metals, such as arsenic and zinc, and organic compound degradation, such as xylan and chitin.

### Data availability.

The complete sequences of the *Microbacterium* sp. Nx66 genome and plasmid have been deposited in DDBJ/EMBL/GenBank under BioProject PRJEB39712 and assembly accession number GCA_904066215. Raw reads have been made available under the same BioProject number with accession numbers ERR4508043 for the MiSeq paired-end reads and ERR4508044 for the ONT long reads. The accession numbers for the annotated sequences of the chromosome and plasmid are LR880474.1 and LR880475.1, respectively.

## References

[1] Hoskisson PA, Fernández-Martínez LT 2018 Regulation of specialised metabolites in Actinobacteria—expanding the paradigm. Environ Microbiol Rep 10:231–238. doi:10.1111/1758-2229.12629. 29457705 PMC6001450

[2] Kamjam M, Sivalingam P, Deng Z, Hong K 2017 Deep sea actinomycetes and their secondary metabolites. Front Microbiol 8:760. doi:10.3389/fmicb.2017.00760. 28507537 PMC5410581

[3] Zhao J, Liu D, Wang Y, Zhu X, Xuan Y, Liu X, Fan H, Chen L, Duan Y 2019 Biocontrol potential of *Microbacterium maritypicum* Sneb159 against Heterodera glycines. Pest Manag Sci 75:3381–3391. doi:10.1002/ps.5546. 31282045

[4] Vílchez JI, Niehaus K, Dowling DN, González-López J, Manzanera M 2018 Protection of pepper plants from drought by *Microbacterium* sp. 3J1 by modulation of the plant’s glutamine and α-ketoglutarate content: a comparative metabolomics approach. Front Microbiol 9:284. doi:10.3389/fmicb.2018.00284. 29520258 PMC5826947

[5] Buss SN, Starlin R, Iwen PC 2014 Bacteremia caused by *Microbacterium binotii* in a patient with sickle cell anemia. J Clin Microbiol 52:379–381. doi:10.1128/JCM.02443-13. 24197889 PMC3911473

[6] Chorost MS, Smith NC, Hutter JN, Ong AC, Stam JA, McGann PT, Hinkle MK, Schaecher KE, Kamau E 2018 Bacteraemia due to Microbacterium paraoxydans in a patient with chronic kidney disease, refractory hypertension and sarcoidosis. JMM Case Rep 5:e005169. doi:10.1099/jmmcr.0.005169. 30619613 PMC6321868

[7] Wongbunmak A, Khiawjan S, Suphantharika M, Pongtharangkul T 2017 BTEX- and naphthalene-degrading bacterium *Microbacterium esteraromaticum* strain SBS1-7 isolated from estuarine sediment. J Hazard Mater 339:82–90. doi:10.1016/j.jhazmat.2017.06.016. 28628786

[8] Erguven GO, Yildirim N 2019 The evaluation of imidacloprid remediation in soil media by two bacterial strains. Curr Microbiol 76:1461–1466. doi:10.1007/s00284-019-01774-w. 31552451

[9] Boubelli S, Chaffai H, Sakaa B, Djidel M, Kherici N 2018 Hydrogeochemical characterization and assessment of the quality of surface waters of Oued Saf-Saf (North-East Algeria). J Bio Env Sci 12:168–178.

[10] Boyer PH, Boulanger N, Nebbak A, Collin E, Jaulhac B, Almeras L 2017 Assessment of MALDI-TOF MS biotyping for *Borrelia burgdorferi* sl detection in Ixodes ricinus. PLoS One 12:e0185430. doi:10.1371/journal.pone.0185430. 28950023 PMC5614582

[11] De Coster W, D'Hert S, Schultz DT, Cruts M, Van Broeckhoven C 2018 NanoPack: visualizing and processing long-read sequencing data. Bioinformatics 34:2666–2669. doi:10.1093/bioinformatics/bty149. 29547981 PMC6061794

[12] Wick RR, Judd LM, Gorrie CL, Holt KE 2017 Unicycler: resolving bacterial genome assemblies from short and long sequencing reads. PLoS Comput Biol 13:e1005595. doi:10.1371/journal.pcbi.1005595. 28594827 PMC5481147

[13] Gurevich A, Saveliev V, Vyahhi N, Tesler G 2013 QUAST: quality assessment tool for genome assemblies. Bioinformatics 29:1072–1075. doi:10.1093/bioinformatics/btt086. 23422339 PMC3624806

[14] Parks DH, Imelfort M, Skennerton CT, Hugenholtz P, Tyson GW 2015 CheckM: assessing the quality of microbial genomes recovered from isolates, single cells, and metagenomes. Genome Res 25:1043–1055. doi:10.1101/gr.186072.114. 25977477 PMC4484387

[15] Wang Q, Garrity GM, Tiedje JM, Cole JR 2007 Naïve Bayesian classifier for rapid assignment of rRNA sequences into the new bacterial taxonomy. Appl Environ Microbiol 73:5261–5267. doi:10.1128/AEM.00062-07. 17586664 PMC1950982

[16] Jain C, Rodriguez-R LM, Phillippy AM, Konstantinidis KT, Aluru S 2018 High throughput ANI analysis of 90K prokaryotic genomes reveals clear species boundaries. Nat Commun 9:5114. doi:10.1038/s41467-018-07641-9. 30504855 PMC6269478

[17] Konstantinidis KT, Tiedje JM 2005 Genomic insights that advance the species definition for prokaryotes. Proc Natl Acad Sci U S A 102:2567–2572. doi:10.1073/pnas.0409727102. 15701695 PMC549018

[18] Mende DR, Sunagawa S, Zeller G, Bork P 2013 Accurate and universal delineation of prokaryotic species. Nat Methods 10:881–884. doi:10.1038/nmeth.2575. 23892899

[19] Médigue C, Calteau A, Cruveiller S, Gachet M, Gautreau G, Josso A, Lajus A, Langlois J, Pereira H, Planel R, Roche D, Rollin J, Rouy Z, Vallenet D 2019 MicroScope—an integrated resource for community expertise of gene functions and comparative analysis of microbial genomic and metabolic data. Briefings Bioinf 20:1071–1084. doi:10.1093/bib/bbx113. PMC6931091 28968784

[20] Lee MD 2019 GToTree: a user-friendly workflow for phylogenomics. Bioinformatics 35:4162–4164. doi:10.1093/bioinformatics/btz188. 30865266 PMC6792077

